# Biallelic TXNIP deficiency is associated with a multisystemic metabolic disease

**DOI:** 10.1016/j.molmet.2026.102414

**Published:** 2026-07-14

**Authors:** Julia-Josefine Scholz, Sharlaine Y.L. Piel, Ioannis Evangelakos, Christian Müller, Daniela Karall, Birgit Reinhart-Steininger, Katja Steinbrücker, Thomas Stulnig, Julia Rohde, Johannes Mayr, Matias Wagner, Ali Biabani, Thomas Mair, Kilian Müller, Hartmut Schlüter, Sören Weidemann, Viktoria Chirico, Christina Weiler-Normann, Christian Hagel, A.S. Knisely, Joerg Heeren, Benedikt Schoser, Christian Kubisch, Maja Hempel, Holger Prokisch, Saskia B. Wortmann, Anna Worthmann, Christian Schlein

**Affiliations:** 1 Institute of Human Genetics, University Medical Center Hamburg-Eppendorf, Hamburg, Germany; 2 Bioinformatics Core, University Medical Center Hamburg-Eppendorf, Hamburg, Germany; 3 Department of Child and Adolescent Health, Division Pediatrics I - Inherited Metabolic Disorders, Medical University of Innsbruck, Innsbruck, Austria; 4 Department of Medicine III and Karl Landsteiner Institute for Metabolic Diseases and Nephrology, Clinic Hietzing, Vienna, Austria; 5 University Children's Hospital, Paracelsus Private Medical University (PMU) Salzburg, Salzburg, Austria; 6 Department of Cardiology, University Heart and Vascular Center Hamburg, University Medical Center Hamburg-Eppendorf, Hamburg, Germany; 7 Institute of Human Genetics, School of Medicine and Health, Technical University of Munich, Munich, Germany; 8 Institute for Neurogenomics, Helmholtz Centre Munich, German Research Center for Health and Environment, Munich, Germany; 9 Section Mass Spectrometry and Proteomics, Center for Diagnostics, University Medical Center Hamburg-Eppendorf, Hamburg, Germany; 10 Institute of Pathology, University Medical Center Hamburg-Eppendorf, Hamburg, Germany; 11 Martin Zeitz Center for Rare Diseases, University Hospital Hamburg-Eppendorf, Hamburg, Germany; 12 I. Department of Medicine, University Hospital Hamburg-Eppendorf, Hamburg, Germany; 13 Institute of Neuropathology, University Medical Center Hamburg-Eppendorf, Hamburg, Germany; 14 Diagnostic and Research Institute of Pathology, Medical University of Graz, Graz, Austria; 15 Department of Biochemistry and Molecular Cell Biology, University Medical Center Hamburg-Eppendorf, Hamburg, Germany; 16 Friedrich-Baur-Institute, Department of Neurology, LMU University Clinic, LMU Munich, Munich, Germany; 17 Institute of Human Genetics, Heidelberg University Hospital, Heidelberg, Germany; 18 Medical Faculty, Heidelberg University, Heidelberg, Germany; 19 Department of Computational Health, Institute of Neurogenomics, Helmholtz Zentrum München, Munich, Germany; 20 now Zentrum für Leber- & Pankreaspathologie, St.-Pölten, Austria

**Keywords:** Inborn errors of metabolism, TXNIP, Mitochondria, Hypertriglyceridemia, Cardiomyopathy

## Abstract

**Objective:**

Thioredoxin-interacting protein (TXNIP) is a protein involved in redox metabolism, but also a key regulator of glucose and lipid metabolism in preclinical models. To date, four patients with biallelic loss-of-function variants in *TXNIP* have been described, presenting with lactic acidosis and variable hypoglycemia, hepatomegaly, developmental delay and seizures. However, the role of TXNIP in human metabolism and its mechanistic effects across different organs are not fully understood.

**Methods:**

We clinically, biochemically and genetically characterized a cohort of six additional individuals with biallelic pathogenic variants in *TXNIP*. Organ specimens from patients and mice were analyzed by gene expression, histology, and lipidomic and proteomic profiling.

**Results:**

We confirmed lactic acidosis as the main clinical sign and added adult-onset cardiomyopathy, skeletal muscle weakness, and dyslipidemia to the extended disease spectrum. Heart, liver and muscle patient specimens showed pathological lipid accumulation, and mechanistic studies uncovered increased fatty acid synthesis markers and complex rearrangements of the lipidome and proteome. In Txnip-deficient mice, restricting dietary carbohydrates partially rescued fatty acid synthesis markers and lipid storage in the heart but led to dyslipidemia.

**Conclusions:**

Our studies show that TXNIP is an important metabolic modifier in cardiac and skeletal muscle as well as in lipoprotein metabolism and that biallelic pathogenic variants in *TXNIP* lead to a pleiotropic disease affecting cellular lipid metabolism in multiple organ systems, with potentially fatal adult-onset cardiomyopathy.

## Highlights

• Biallelic loss of *TXNIP* results in a complex disease with a potentially life-threatening metabolic disease in humans.
• TXNIP is an important regulator of triglyceride-rich lipoprotein metabolism and cellular triglyceride storage.
• Loss of TXNIP leads to increased intracellular lipid storage via elevated *de novo* lipogenesis in humans and mice.
• Lowering *de novo* lipogenesis by carbohydrate restriction is a potential therapeutic approach.

## 1 Introduction

Thioredoxin-interacting protein (TXNIP) or Thioredoxin Binding Protein-2 (TBP-2) was first identified in vitamin-D stimulated HeLa-cells and therefore initially named vitamin D3-upregulated protein 1 [1]. Upon the discovery of its role as a negative regulator of Thioredoxin (TXN) in redox metabolism during oxidative stress [2,3], it was renamed. Besides numerous redox-state dependent functions, including promotion of apoptosis and inflammation [[4], [5], [6]], TXNIP also exerts several redox-independent roles. These include the regulation of cell growth, carcinogenesis, cardiac function and metabolism [4]. In the latter it critically regulates glucose- and lipid homeostasis [7].

TXNIP expression is foremost induced by glucose through a carbohydrate response element in its promoter. This creates a negative feedback loop for cellular glucose homeostasis [8] in which TXNIP acts as an adapter protein supporting endocytosis of glucose transporter 4 (GLUT4, encoded by *SLC2A4*) and other glucose transporters [9,10]. In the fed state, with sufficient intracellular glucose concentrations, this limits glucose influx. In the fasted state, reduced TXNIP expression permits glucose influx. Consequently, loss of TXNIP results in uncontrolled glucose influx and hypoglycemia [11]. On the other hand, hyperglycemia induces TXNIP expression [[12], [13], [14]] and TXNIP was shown to induce beta-cell apoptosis when those conditions persist, thus fueling the vicious circle of diabetes onset and its progression [12,14,15]. Several studies reported increased TXNIP expression in the context of metabolic disorders such as insulin resistance, type 2 diabetes, and impaired glucose tolerance, and proposed its pharmacological inhibition as a promising target in combating these metabolic comorbidities [16]. TXNIP is also studied in the context of cardiometabolic diseases, as TXNIP overexpression promotes myocardial infarction, atherosclerosis, cardiomyopathy, and cardiotoxicity, while genetic deletion or pharmacological inhibition of TXNIP inhibits onset and/or development of these conditions in preclinical models [17].

*Txnip*-knockout (KO) mice as well as the mouse-line HcB-19/Dem (HcB-19, “Hyplip”), which carries a spontaneous nonsense mutation in *Txnip* [18] and was originally studied for hypertriglyceridemia and hypercholesterolemia, present profound metabolic alterations including hyperinsulinemic hypoglycemia and hyperlipidemia. Mechanistically, the latter was found to be attributed to elevated hepatic *de novo* lipogenesis (DNL), a process where non-lipid precursors (e.g. carbohydrates) are physiologically used for the endogenous synthesis of fatty acids. Increased hepatic DNL in *Txnip-*KO mice was demonstrated by increased lipogenic gene expression, fatty acid synthase activity and radioactively traced fatty acid synthesis [[19], [20], [21], [22], [23], [24]]. Interestingly, one study in *Txnip*-KO mice neither confirmed hyperinsulinemia and hyperlipidemia, nor elevated hepatic DNL [25], indicating that TXNIP's action and lipid-metabolism deficiency may show incomplete penetrance or variable expressivity. Lactate elevation, on the other hand, was observed in all studies. Investigations of cardiac manifestations of TXNIP deficiency revealed altered substrate utilization, underlined by increased glucose uptake [26] and glycolysis, but decreased glucose-driven mitochondrial oxidation, leading to compensatory increased fatty acid oxidation [27]. *Txnip*-KO mice display largely normal baseline cardiac structure and function but, interestingly, exhibit significant cardioprotection against myocardial infarction [28], ischemia-reperfusion injury [29], and high-fat diet-induced cardiomyopathy [30].

However, detailed clinical characterization of human TXNIP deficiency is limited, and human and murine phenotypes remain to be correlated. In one case report, three siblings with biallelic pathogenic variants in *TXNIP* had lactic acidosis and low serum methionine. One child presented hypoglycemia and elevated serum transaminase activity, hinting at hepatic involvement [31]. Recently, an additional (fourth) patient case was published within a study focusing on TXNIP mediation of LAT1/SLC7A5 (amino acid transporter) endocytosis to limit amino acid uptake in cells entering quiescence [32]. However, cardiometabolic (pre-) clinical or mechanistic investigations were not performed in either report.

Using in-depth clinical phenotyping and preclinical models, we have expanded the spectrum of *TXNIP*-associated human disease, adding an association with cardiac and muscle involvement represented by adult-onset cardiomyopathy and early-onset skeletal muscle weakness. We were able to confirm lactic acidosis as the main clinical sign of *TXNIP*-associated disease and found developmental delay and seizures to be variable symptoms. Importantly, we found hypertriglyceridemia to be a new feature of *TXNIP*-associated disease. Mechanistically, we found that biallelic variants – nonsense variants, missense variants, or a 334 kb microdeletion in 1q21.1 including the *TXNIP* gene locus – result in high levels of fatty acid synthesis markers in humans, as also seen in *Txnip*-KO mice. Therapeutic investigations in preclinical models revealed elevated fatty acid synthesis and pathological lipid storage in the hearts of *Txnip*-KO mice, partially reversible when a low-carbohydrate diet was introduced. This is in line with our clinical observation, as sugar restriction led to increased self-reported well-being in one patient, whereas a ketogenic diet resulted in severe hypertriglyceridemia in another patient, highlighting that dietary sugar and fat intake may modify the clinical phenotype in TXNIP deficiency. In sum, we describe TXNIP as an important regulator of cellular, systemic, and multi-organ lipid metabolism relevant for human and murine cardiovascular health.

## 2 Results

### 2.1 Clinical characterization of individuals with pathogenic biallelic variants in *TXNIP*

**Figure 1:**
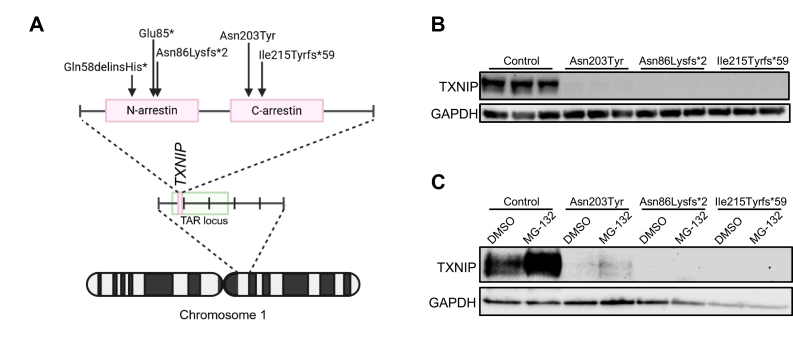
**Biallelic variants in *TXNIP* eliminate TXNIP from patient-derived fibroblasts**. (A) Chromosomal location and structure of *TXNIP* with C- and N-terminal arrestin domains. Arrows indicate variant position and genes included in the TAR deletion. *Created in BioRender. Schlein, C. (2026) https://BioRender.com/ ma6iw36.* (B) Western blot, lysates, control fibroblasts (n = 3) and TXNIP Asn203Tyr, Asn86Lysfs∗2, and Ile215Tyrfs∗59 fibroblasts (each n = 3), against TXNIP and GAPDH (loading control). (C) Western blot, lysates as above, against TXNIP and GAPDH after treatment with 10 mM MG-132.

As TXNIP function is associated with glycemic control and lipid homeostasis in preclinical models, we were interested in the role of TXNIP in human lipid metabolism. For this purpose, we identified humans with biallelic variants in *TXNIP* (NM_006472.6), performed in-depth clinical characterization of these individuals (Table 1, individuals #1–6, Figure 1A; see Supplementary information for medical history), and compared their clinical phenotypes to those of the previously described three siblings homozygous for the p.Gln58delinsHis∗ variant [31] (Table 1, Figure 1A, individuals #8–10) and one individual homozygous for the p.Ile215Tyrfs∗59 variant [32] (Table 1, Figure 1A, individual #7). The six additional individuals with biallelic variants were from five independent families. Two families were recruited within the German/Austrian network for mitochondrial disorders (mitoNET), two families presented individually at medical-genetic policlinics with cardiomyopathy, and one family was studied because genetic disease was suspected in their infant during a hospitalization (Table 1). In this cohort, two individuals were children (#3, and #5) and four were adults (individuals #1, #2, #4, and #6).

**Table 1:** **Clinical table of individuals with biallelic variants in *TXNIP*.** Ten patients, six novel (from five families) and four reported, are included. The most frequent features of disease were lactic acidosis (10/10), muscular hypotonia (6/9), hypoglycemia (5/9) and hypertriglyceridemia (4/9). Cardiomyopathy (adult-onset) was present in three patients, leading to death in two (denoted by †). Patients #1 and #2 are brothers; patients #8, #9 and #10 are siblings. LoF, loss of function; DD/ID, developmental delay/intellectual disability.

Individual	**#1**	**#2**	**#3**	**#4**	**#5**	**#6**	**#7**	**#8**	**#9**	**#10**	
Variant	p.Glu85∗; TARdel		p.Asn86Lysfs∗2	p.Asn86Lysfs∗2	p.Asn86Lysfs∗2	p.Asn203Tyr	p.Ile215Tyrfs∗59 Kahlhofer et al. [32]	p.Gln58delinsHis∗ Katsu-Jiménez et al. [31]			
	c.253G>T; [GRCh38] del(1)(q.21.1) chr1:g.145687562-146021675del		Homozygous c.257dupA	Homozygous c.257dupA	Homozygous c.257dupA	Homozygous c.607A>T	Homozygous c.642_643insT	Homozygousc.174_175delinsTT			
Mode of pathogenicity	LoF	LoF	Frameshift	Frameshift	Frameshift	Missense	Frameshift	LoF	LoF	LoF	
Sex	Male	Male	Male	Female	Male	Male	Male	Male	Female	Male	
Age at first presentation	21 years	33 years	Neonatal	26 years	Neonatal	18 years	Neonatal	Neonatal	Neonatal	Neonatal	
Age at last evaluation	47 years	33 years †	6 years	30 years	5 months	35 years †	10 years	13 years [31]	9 years [31]	4 years [31]	
Lactic acidosis	+	+	+	+	+	+	+	+	+	+	10/10
Hypertriglyceridemia	+	?	+	–	–	+	+	–	–	–	4/9
Muscular hypotonia	+	?	+	–	+	+	+	–	–	+	6/9
Hepatomegaly	–	?	+	–	–	+	–	+	?	?	3/7
Arterial hypertension	+	?	–	+	–	+	–	?	?	?	3/6
Hypoglycemia	+	?	–	+	–	+	+	+	–	–	5/9
Developmental delay/intellectual disability	–	–	+	–	+	–	+	–	–	+	4/10
Seizures	–	–	–	–	–	+	+	–	–	–	2/10
Cardiomyopathy	+	+	–	–	–	+	–	–	–	–	3/10

Patients #3, #4, and #5 had homozygous *TXNIP* frameshift variants (Table 1, Figure 1A) and #6 had a homozygous *TXNIP* missense variant. Two brothers, #1 and #2, had a heterozygous 334 kb microdeletion in 1q21.1 within the critical region for autosomal-recessive thrombocytopenia-absent radius syndrome (TAR) including deletion of the *TXNIP* gene locus, in combination with a nonsense variant on the non-deleted *TXNIP* allele. Individual #2 died years before medical-genetic evaluation, with testing performed *post mortem*.

The main clinical feature (*cf.* Supplementary information) in the three individuals described by Katsu-Jiménez et al. [31], the single individual described by Kahlhofer et al. [32] and the six newly studied individuals was lactic acidosis (present in 10/10). Muscular hypotonia (6/9 evaluated individuals), hypoglycemia (5/9), hypertriglyceridemia (4/9), arterial hypertension (3/6), hepatomegaly (3/7), adult-onset cardiomyopathy (3/4 adult individuals or 3/10 of all individuals), developmental delay (4/10), and seizures (2/10) were also observed. Lactic acidosis was present on every routine clinical-chemistry assessment; although hypertriglyceridemia was repeatedly seen, triglyceride values were occasionally within the reference range in some individuals. Hypoglycemia occurred mostly in fasting periods and was asymptomatic in most individuals, but did not occur when individual #1 followed a sugar-free diet. Three of four adults had severe cardiomyopathy. Individual #2 died aged 33 due to cardiomyopathy-associated complications one week after cardiomyopathy was diagnosed. Individual #6 died aged 35 after an anesthesia-associated cardiac arrest. He had severe hypertriglyceridemia (plasma triglycerides >1600 mg/dL, ref. < 150 mg/dL). Individual #7, despite normolipidemic at birth, developed hypertriglyceridemia (≤364 mg/dL). Individual #3 had mild hypertriglyceridemia, which was exacerbated on a ketogenic diet (>10,000 mg/dL).

Impaired function of TXNIP thus appears to affect especially skeletal and cardiac muscle- as well as liver-related functions, deranging metabolic homeostasis and leading to dyslipidemia, hypoglycemia and lactic acidosis.

### 2.2 Biallelic variants in *TXNIP* eliminate TXNIP from patient-derived fibroblasts

To assess the impact of different *TXNIP* variants on TXNIP protein abundance (Figure 1A), we performed western blot analysis of cell lysates of cultured patient-derived fibroblasts. The results confirmed that TXNIP was not detectable in lysates of fibroblasts derived from individuals with a homozygous p.Asn86Lysfs∗2 or a p.Ile215Tyrfs∗59 variant in *TXNIP* (Figure 1B). However, in the fibroblasts derived from individual #6, with a homozygous p.Asn203Tyr variant in *TXNIP*, a residual minimal fraction of TXNIP was detected (Figure 1B). We hypothesized that the protein is translated but rapidly degraded via the ubiquitin-proteasome pathway during protein quality control. To investigate this, we incubated patient-derived fibroblasts with the proteasome inhibitor MG-132 and performed western blot analysis. An increased signal was detected in fibroblasts from individual #6, indicating that the encoded protein undergoes rapid posttranslational degradation (Figure 1C), thereby explaining the sparse signal under standard culture conditions. These results indicate functional relevance of the analyzed *TXNIP* variants. Lipidome analyses (“lipidomics”) from patient-derived fibroblasts from individuals #4, 6, and 7 showed minor alterations, mostly distinguished from controls by low lactosyl-ceramide levels (LCER) in random forest plot analysis (Metaboanalyst [33]). Low levels of LCER, a lipid class involved in redox stress [34] (Supplementary Fig.1a, b), might indicate that TXNIP deficiency reduces redox stress [35,36] in this metabolically rather inactive setting. However, as the clinical phenotype mostly involved metabolically active tissues and the circulation, we further focused on metabolically active organs and related models for further clinical and preclinical investigations.

### 2.3 Plasma profiling in individuals with biallelic *TXNIP* variants and in *Txnip*-KO mice

**Figure 2:**
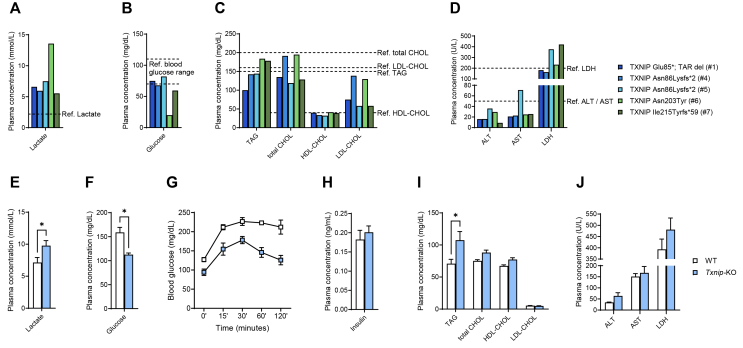
**Plasma profiling in individuals with biallelic *TXNIP* variants and in *Txnip*-KO mice**. (A–D): Plasma profiling in individuals with *TXNIP* variants yielding TXNIP p.Glu85∗; TAR del (individual #1), p.Asn86Lysfs∗2 (individual #4 and #5), p.Asn203Tyr (individual #6), and p.Ile215Tyrfs∗59 (individual #7), with correlating reference values. Plasma (A) lactate, (B) glucose, (C) lipid profile including triglycerides (TAG), total cholesterol (total CHOL), HDL-cholesterol (HDL-CHOL) and LDL-cholesterol (LDL-CHOL), (D) activities of liver transaminases alanine aminotransferase (ALT) and aspartate aminotransferase (AST) and of lactate dehydrogenase (LDH). (EJ): Plasma profiling in *Txnip*-KO mice and WT littermates. Plasma (E) lactate, (F) glucose, (G) oral glucose tolerance, (H) insulin, (I) lipid profile including triglycerides (TAG), total cholesterol (total CHOL), HDL-cholesterol (HDL-CHOL) and LDL-cholesterol (LDL-CHOL), (J) activities of liver transaminases alanine aminotransferase (ALT) and aspartate aminotransferase (AST) and lactate dehydrogenase (LDH). (E-F, H-J) WT mice n = 11, *Txnip*-KO mice n = 10. (G) WT mice n = 5, *Txnip*-KO mice n = 7.

To assess the systemic metabolic impact of TXNIP deficiency, we analyzed plasma of individuals with biallelic variants in *TXNIP* and *Txnip*-KO mice. In human plasma (n = 5, individuals #1, #4, #5, #6 and #7), we confirmed hyperlactatemia in all individuals (Figure 2A) and hypoglycemia in 3/5 individuals (Figure 2B), serving as clinical surrogate markers for impaired metabolic function. Standard lipid profiling showed above-reference triglycerides in 2/5 individuals and below- or near-reference total cholesterol, HDL-cholesterol, and LDL-cholesterol in all individuals (Figure 2C). Liver enzymes ALT and AST were below reference values except in individual #5; whereas LDH as a lactate-metabolizing enzyme, was measured above (individual #5, 6, and 7) or near reference (individual #1 and 4) (Figure 2D). Plasma profiles in humans with biallelic *TXNIP* variants were mirrored in a mouse model: Elevated plasma lactate and decreased plasma glucose levels were also observed in plasma of *Txnip*-KO mice (Figure 2E, F) accompanied by elevated ketone body values (Supplementary Fig.2a). In line with published results [27,37], oral glucose tolerance in the murine model revealed that *Txnip*-KO mice show glucose tolerance higher than wildtype (WT) littermates (Figure 2G). Insulin levels were comparable with those in WT mice (Figure 2H). Triglyceride levels were elevated, while cholesterol levels differed only slightly from those in WT littermates (Figure 2I). Values for liver enzymes ALT and AST were not altered, and LDH as a lactate-metabolizing enzyme was elevated by trend (p = 0.15) in *Txnip*-KO mice (Figure 2J).

### 2.4 Complex lipidomic rearrangements in plasma of individuals with biallelic variants in *TXNIP*

**Figure 3:**
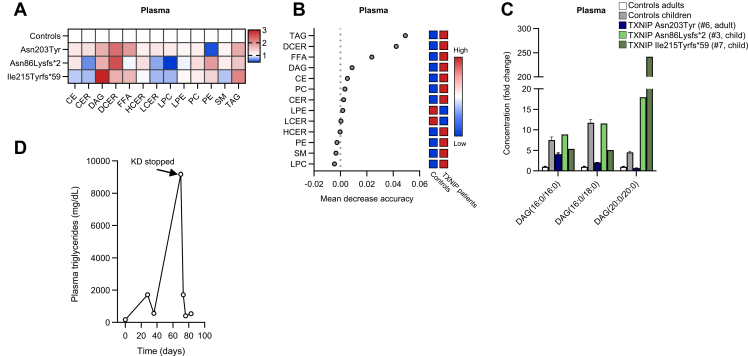
**Complex lipidomic rearrangements in plasma of individuals with biallelic variants in *TXNIP***. (A) Heatmap of plasma lipid concentration-fold changes, patients with TXNIP variants Asn203Tyr, Asn86Lysfs∗2, and Ile215Tyrfs∗59 (each n = 1) versus controls (n = 9). (B) Random forest plot of lipid class concentration-folds, plasma of patients (n = 3) versus controls (n = 12), as mean decrease accuracy. (C) Plasma concentration-fold changes of diglycerides (16:0/16:0), (16:0/18:0) and (20:0/20:0) in individuals with *TXNIP* variants yielding TXNIP p.Asn203Tyr, TXNIP p.Asn86Lysfs∗2 and TXNIP p.Ile215Tyrfs∗59 (n = 1 each) versus adult (n = 9) and children controls (n = 3). (D) Plasma triglyceride measurements before, during, and after start and stop of ketogenic diet, individual #3.

As clinical data and/or plasma profiling revealed hypertriglyceridemia in a subset of individuals with biallelic variants in *TXNIP* and in *Txnip*-KO mice*,* we performed targeted lipidome analysis of patient plasma and age- and sex-matched healthy controls to further characterize the impact of *TXNIP* dysfunction on human lipid metabolism. We detected over 800 lipid species from 13 lipid classes. Random forest plotting of lipid class concentrations (Metaboanalyst [33]) revealed that triglycerides (TAG), diglycerides (DAG), and free fatty acids (FFA) were the top lipid classes associated with biallelic pathogenic variants in *TXNIP*, distinguishing individuals with such variants from their matched controls (Figure 3A). Interestingly, biallelic variants in *TXNIP* were also associated with higher levels of dihydroceramides (DCER), a lipid class important in cellular stress responses as plasma levels of DCER have been linked to metabolic disease [38] (Figure 3A, B). Elevations in fasting TAG were mild to moderate (around twice those in controls), but were detected in all three available patient-derived plasma samples. Additionally, levels of DAG were higher in patients than in controls, suggesting either hydrolysis of TAG or a decreased hepatic fatty acid re-esterification rate and subsequent release of immature complex lipids into plasma lipoproteins. Speciation of DAG showed higher abundance of saturated lipids, such as those with two palmitate residues, DAG (16:0/16:0); with palmitate and stearate, DAG (16:0/18:0); or the rare di-arachidin, DAG (20:0/20:0) (Figure 3C). Overall, in light of the higher saturated fatty acid residues, and in line with published literature in animal models [20,21,24], the question arose whether elevated TAG levels result from enhanced endogenous fatty acid synthesis (a major source of saturated fatty acids [18]), or from higher cellular lipid uptake.

Of note, individual #3 developed severe hypertriglyceridemia (peak >10,000 mg/dL, Figure 3D) under a ketogenic diet, which rapidly reversed after its discontinuation. This indicates that diet may be a major regulator of hypertriglyceridemia in individuals with biallelic variants in *TXNIP*.

### 2.5 Elevated liver fatty acid synthesis in an individual with biallelic variants in *TXNIP*

As hepatomegaly was noted in some individuals with biallelic variants in *TXNIP* and as *Txnip*-KO mice have increased hepatic DNL contributing to hepatic steatosis and hyperlipidemia [20,21,24], we investigated the effects of human TXNIP deficiency on the liver.

**Figure 4:**
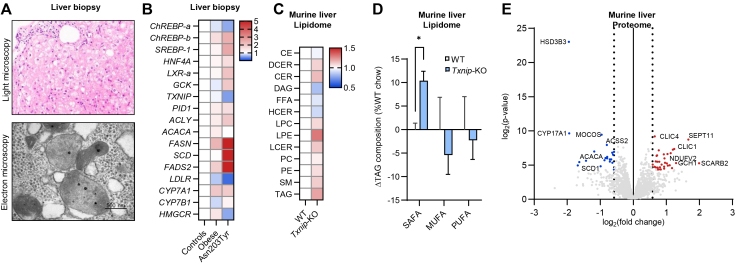
**Elevated liver fatty acid synthesis in an individual with biallelic variants in *TXNIP***. (A) Light micrograph (H&E) and transmission electron micrograph image of liver tissue, individual #6, showing glycogen accumulation and abnormal mitochondrial structure with irregular cristae. (B) Heatmap showing gene expression, liver of TXNIP Asn203Tyr patient (n = 1) *vs.* healthy controls (n = 8) and obese individuals (n = 9), red indicating higher and blue lower gene expression. (C) Murine liver lipidome, lipid concentration-fold changes in *Txnip*-KO mice and WT littermates. (D) TAG lipid class composition differentiating saturated fatty acids (SAFA), monounsaturated fatty acids (MUFA) and polyunsaturated fatty acids (PUFA), liver lipidome, *Txnip*-KO mice and WT littermates, displayed as % change compared to WT. (E) Volcano plot, liver proteome, *Txnip*-KO mice and WT littermates. CE: cholesteryl esters, CER: ceramides, DAG: diglycerides, DCER: dihydroceramides, FFA: free fatty acids, HCER: hexosylceramides, LCER: lactosylceramides, LPC: lysophosphatidylcholine, LPE: lysophosphatidylethanolamine, PC: phosphatidylcholine, PE: phosphatidylethanolamine , SM: sphingomyelins, TAG: triglycerides.

Individual #6 underwent diagnostic liver biopsy due to elevated serum transaminase activity. Both light microscopy (hematoxylin/eosin, H&E; periodic acid-Schiff technique, PAS) and transmission electron microscopy (TEM) of the liver-biopsy specimen revealed a moderate accumulation of glycogen together with the presence of lipid droplets (Figure 4A). Further, TEM uncovered structural irregularities in mitochondria including few and irregular cristae (Figure 4A). In tissue that had been preserved, gene-expression analysis showed higher expression of genes related to DNL, including *FASN* and *SCD* (both 5-fold), as well as of key lipogenic transcription factors such as *ChREBP-beta* and *SREBP1c* (Figure 4B). Transcriptional activation of lipogenesis pathways was higher than in obese patient controls (Figure 4B), suggesting a condition already connected with high lipogenesis [39]. Patient material was insufficient for further lipidomic analyses. Accordingly, livers from WT and *Txnip*-KO mice were studied. Hepatic lipidomic analysis revealed higher levels of CER (Figure 4C) in *Txnip*-KO mice than in WT littermates, a lipid class increased in inflammation [40] and in insulin resistance [41]. Further, compositional analysis of TAG revealed a higher proportion of saturated fatty acids, potentially indicating hepatic endogenous fatty acid synthesis activity in *Txnip*-KO mice higher than that in WT (Figure 4D). To gather mechanistic insights into the hepatic lipidomic fingerprint of the *Txnip*-KO mice, we further performed untargeted proteomic analysis of the tissues (Figure 4E). A volcano plot analysis revealed low levels of enzymes involved in steroid metabolism (HSD3B3, CYP17A1) but no clear pattern resembling the gene-expression phenotype seen in individual #6. Despite compositional changes supporting high endogenous fatty acid and CER synthesis (Figure 4C), hepatic protein expression in the fatty acid synthesis pathway (ACACA, ACSS2, SCD1) and in CER synthesis (CERS2) was reduced or unchanged in *Txnip*-KO mice, indicating regulation independent of hepatic protein expression.

### 2.6 TXNIP as a regulator of metabolic pathways in human and murine skeletal muscle

**Figure 5:**
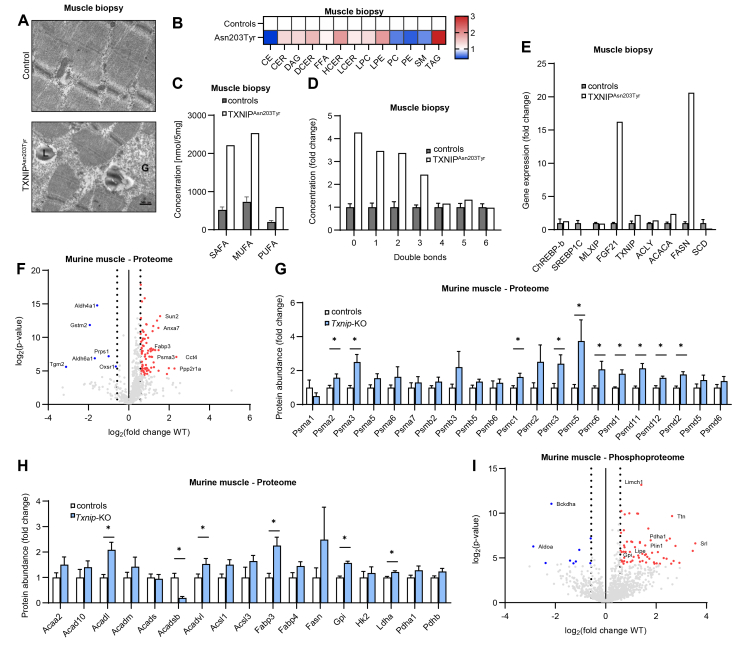
**TXNIP as a regulator of metabolic pathways in human and murine skeletal muscle**. (A) TEM images, control muscle tissue and muscle biopsy specimen, individual #6. G: glycogen; L: lipid droplets. (B) Heatmap showing muscle lipid class concentration-fold changes, muscle biopsy, individual #6 (n = 1) versus controls (n = 3). (C) Concentrations of saturated fatty acids (SAFA), monounsaturated fatty acids (MUFA) and polyunsaturated fatty acids (PUFA), muscle biopsy specimen, individual #6 (n = 1) versus controls (n = 3). (D) Fold change, concentration of double bonds in fatty acids, muscle biopsy specimen, individual #6 (n = 1) versus controls (n = 3). (E) Gene expression-fold changes, key regulators in muscle biopsy specimen, individual #6 (n = 1) versus healthy controls (n = 3). (F) Volcano plot and (G–H) protein abundance (proteomics), muscle of *Txnip*-KO mice and of WT littermates. (I) Volcano plot, phosphoproteomics, muscle of *Txnip*-KO mice and of WT littermates.

Individual #6 also underwent diagnostic muscle biopsy because of muscle weakness and chronically elevated creatine kinase levels. TEM of skeletal muscle revealed accumulation of lipid droplets and glycogen deposits (Figure 5A) more abundant than in controls, as observed for liver (Figure 4A). We assessed the composition of the lipid deposits by targeted lipidome analyses. In line with the fact that TAG predominate among stored lipids in cellular lipid droplets, we found high levels of TAG in the muscle of individual #6. Further, levels of hexosylceramides (HCER), DCER, and CER were slightly elevated, whereas levels of cholesteryl esters, phosphatidylcholine, phosphatidylethanolamine, and sphingomyelin were lower (Figure 5B). These findings indicated robust and complex remodeling of the muscle. When we analyzed the concentrations of specific lipid species, TAG containing saturated or monounsaturated fatty acids were particularly elevated (Figure 5C). The absolute concentrations of saturated, monounsaturated, and polyunsaturated fatty acids were elevated, with saturated and monounsaturated fatty acids as the main fractions.

Plotting of double bonds of fatty acid residues within the TAGs showed that lipid accumulation comprised TAG species with low levels of desaturation or without desaturation (Figure 5D). This composition exists in tissues with very high fatty acid synthase activity, which prevents the use of polyunsaturated fatty acids for complex lipids [18]. Gene-expression analysis in the muscle of individual #6 found elevated expression of *ACACA* (2.3-fold) and *FASN* (20-fold), key enzymes in the DNL pathway, as well as of stress-related *FGF21* (16-fold) (Figure 5E). However, expression levels of canonical transcription factors of endogenous fatty acid synthesis in skeletal muscle, such as *MLXIP* or *SREBP1c*, were not altered. Moreover, expression of *SCD*, encoding stearoyl-CoA 9-desaturase (SCD), was lower. SCD catalyzes mono-unsaturated fatty acid production and is usually transcriptionally co-expressed with *FASN*, indicating that the increase in MUFAs might also be partially driven by uptake of exogenous lipids from the circulation.

To generalize these findings obtained in a biopsy specimen from a single patient and to study this new phenotype in mice, we performed untargeted proteome analyses (“proteomics”) of skeletal muscle tissue from wild-type and *Txnip*-KO mice (Figure 5F–H). In *Txnip*-KO murine muscle, proteasome subunits and regulators of the PSMA/PSMB/PSMC/PSMD families were substantially elevated, indicating possible derangement of the proteasomal machinery (Figure 5G). Lipid metabolism, which was widely affected in humans, showed a trend towards higher pathway translation in *Txnip*-KO mice, such as higher ACADL, ACADVL, and FABP3 and a trend to higher FASN (Figure 5H). Phosphoproteome analyses (“phosphoproteomics”) of skeletal muscle of *Txnip*-KO mice, conducted to investigate underlying signaling pathways, indicated elevated levels of lipid-droplet–associated proteins (PLIN1, LIPE), and reduced levels of 2-oxoisovalerate dehydrogenase subunit alpha (BCKDHA), active in branched-chain amino acid metabolism, and of pyruvate dehydrogenase (PDHA1) in comparison to WT (Figure 5I). Thus, it was evident that TXNIP affected multiple anabolic and catabolic pathways in skeletal muscle, such as proteostasis and lipid metabolism.

### 2.7 TXNIP deficiency leads to storage lipid accumulation in human and murine heart tissue

**Figure 6:**
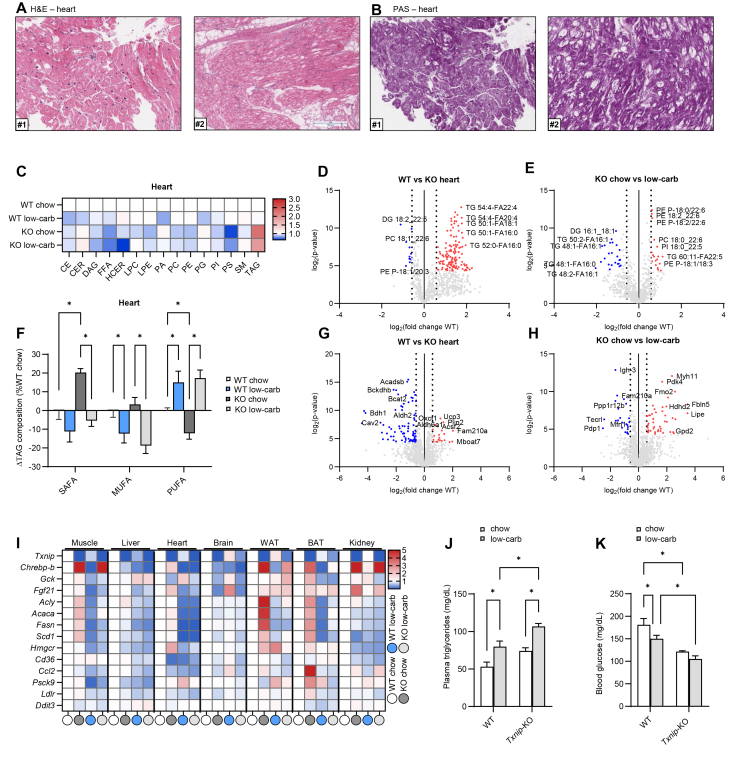
**TXNIP deficiency leads to storage lipid accumulation in human and murine heart tissue; low-carbohydrate diet reduces fatty acid synthesis but leads to hyperlipidemia in *Txnip*-KO mice**. (A), (B) Light micrographs, myocardial biopsy specimens, individuals #1 and #2; H&E, (A), and PAS, (B). (C) Heat map and (D, E) volcano plots and (F) TAG lipid class composition differentiating saturated fatty acids (SAFA), monounsaturated fatty acids (MUFA), and polyunsaturated fatty acids (PUFA), heart tissue lipidomes, *Txnip*-KO and WT mice on chow or low-carbohydrate diet (n = 5). (G, H) Heart tissue proteomes, *Txnip*-KO and WT mice on chow or low-carbohydrate diet (n = 5). (I) Heatmaps, gene expression, muscle, liver, heart, brain, white adipose tissue (WAT), brown adipose tissue (BAT), and kidney, *Txnip*-KO and WT mice on chow or low-carbohydrate diet (n = 5). (J) Plasma triglycerides, *Txnip*-KO and WT mice on chow or low-carbohydrate diet (n = 5). (K) Plasma blood glucose, *Txnip*-KO and WT mice on chow or low-carbohydrate diet (n = 5). CE: cholesteryl esters, CER: ceramides, DAG: diglycerides, DCER: dihydroceramides, FFA: free fatty acids, HCER: hexosylceramides, LPC: lysophosphatidylcholine, PA: phosphatidic acid, PG: phosphatidyl glycerol, PI: phosphatidyl inositol, PS: phosphatidyl serine, LPE: lysophosphatidylethanolamine, PC: phosphatidylcholine, PE: phosphatidylethanolamine , SM: sphingomyelins, TAG: triglycerides.

Three of four adults with biallelic variants in *TXNIP* had severe cardiomyopathy, being clinically fatal in two of the cases (see medical history in Supplementary information). Light microscopy of H&E- (Figure 6A) and PAS- (Figure 6B) stained myocardial biopsy specimens from individuals #1 and #2 revealed interstitial fibrosis in #1 and myocyte hypertrophy with severe steatosis in #2; the biopsy in #2 was conducted a few days prior to demise.

Interestingly, a detailed anamnesis found that for many years, individual #1 had strictly limited sugar intake for several weeks at a time, which he reported to have improved his sense of well-being and muscular fitness (see Supplementary information). We hypothesized that this phenomenon might be explained by reductions in cellular fatty acid synthesis and sought to mimic this in our preclinical model, studying WT and *Txnip*-KO mice on chow (standard) and low-carbohydrate diets. Indeed, lipidomics of heart tissues revealed high TAG lipid storage in the hearts of *Txnip*-KO mice upon chow diet (Figure 6C). Two weeks of a low-carbohydrate diet reduced heart TAG storage from that seen with chow diet (∼20%, *p* < 0.05; Figure 6C), which indicates that dietary interventions can lower TAG in heart muscle of *Txnip*-KO mice. Of note, TAG content in heart tissue of WT mice did not change in response to a low-carbohydrate diet (Figure 6C). To speculate that deregulated lipid metabolism in part underlies potentially lethal cardiac pathophysiology in humans is tempting, especially as the myocyte hypertrophy with steatosis seen on myocardial biopsy in individual #2 was more severe than in his brother (individual #1) who periodically followed a low-/no-carbohydrate diet (Figure 6A,B). Individual #2 reportedly did not follow a special diet.

Volcano plotting of lipid species indicated that palmitate species, but also MUFA and PUFA triglycerol species, were “top hits” (Figure 6D). However, whilst a low-carbohydrate diet lowered levels of palmitate and palmitoleate TAG species in *Txnip*-KO mice (Figure 6E), it elevated plasmalogen levels, members of an anti-oxidative lipid class highly abundant in heart tissue [42], and of polyunsaturated species of phosphatidylethanolamine and phosphatidylcholine (Figure 6E). To compare saturated and unsaturated lipid species quantitatively, TAG composition was plotted for the different groups (Figure 6F). On chow diet, *Txnip*-KO hearts contained more saturated fatty acids and fewer PUFAs than the WT tissues. These differences vanished substantially on a low-carbohydrate diet, indicating that a dietary regimen focused on reducing endogenous fatty acid synthesis might be a promising approach to reduce lipid storage and to normalize lipid composition. Proteomic analysis of hearts of *Txnip*-KO mice and WT littermates revealed a complex shift in TXNIP deficiency, with increased levels of lipid anabolism marker PLIN2 and reduced levels of markers of branched-chain amino acid metabolism (Figure 6G). Interestingly, a low-carbohydrate diet induced LIPE in *Txnip*-KO mice, indicating that TAG hydrolysis might be a mechanism by which a low-carbohydrate diet can contribute to reducing lipid storage (Figure 6H).

### 2.8 Low-carbohydrate diet reduces fatty acid synthesis but leads to hyperlipidemia in *Txnip*-KO mice

Gene-expression analysis was used to screen a set of mouse organs for markers of fatty acid synthesis. This revealed that *Txnip*-KO mice on chow diet exhibited higher expression of markers associated with fatty acid synthesis, such as the lipogenic transcription factor carbohydrate responsive element binding protein isoform beta (ChREBP-beta) [43], an alternative splicing product encoded by *Mlxipl* and a well-described interaction partner of TXNIP [44], in skeletal muscle, heart, white adipose tissue, brown adipose tissue, and kidney (Figure 6I). This was accompanied by higher *Fasn* expression in skeletal muscle, heart, and white and brown adipose tissues (Figure 6I). Additionally, expression of the glycogen synthesis marker *Gck* was notably higher, particularly in the liver, where it represents the predominant isoform, alongside hexokinase 1 and hexokinase 2, which are the main isoforms in other organs (Figure 6I). A low-carbohydrate diet effectively lowered *Fasn* expression in skeletal muscle, heart, and white and brown adipose tissues and lowered ChREBP-beta expression in all organs except kidney and skeletal muscle; in skeletal muscle, instead of ChREBP-beta, *Mlxip,* encoding MondoA*,* is the main lipogenic transcription factor (Figure 6I). Overall, based on gene expression of the surrogate marker *Fasn*, a low-carbohydrate diet may be a route to reducing pathologically elevated fatty acid synthesis and, to some extent, glycogen synthesis in *Txnip*-KO mice.

As fatty acid synthesis is hypothesized to be potentially linked to the phenotype of elevated plasma TAG levels, we hypothesized that lower fatty acid synthesis might result in lower plasma TAG levels. However, a low-carbohydrate diet resulted in higher plasma TAG levels in both WT and *Txnip*-KO mice (Figure 6J). This suggests that, in *TXNIP* deficiency, not only high fatty acid synthesis but also other mechanisms may drive increases in plasma TAG, such as impaired vascular catabolism of TAG-rich lipoproteins or increased dietary lipid intake associated with a low-carbohydrate diet.

With hypoglycemia being a recurrent but not universal finding in the reported individuals with *TXNIP* variants and literature-reported *Txnip*-KO mice, we also investigated fasting blood glucose levels. First, while fed a chow diet, *Txnip*-KO mice indeed displayed lower fasting blood glucose levels than WT mice (WT mean: 181 mg/dL, *Txnip*-KO mean: 122 mg/dL) (Figure 6K). Second, while fed a low-carbohydrate diet, both groups had lower fasting blood glucose levels (WT mean: 150 mg/dL, *Txnip*-KO mean: 105 mg/dL) than when fed a chow diet (Figure 6K). Of note, *Txnip*-KO mice in our study never displayed fasting hypoglycemia regardless of diet (hypoglycemia defined as blood glucose <60 mg/dL; lowest value measured in low-carbohydrate-group 88 mg/dL).

## 3 Discussion

TXNIP, a multifunctional molecule, is not only a regulator in the redox system but also important in glucose and lipid homeostasis. In human disease, it is associated with the pathogenesis of diabetes mellitus and studied as a therapeutic target for hyperglycemia and associated diseases. Our work highlights that biallelic pathogenic variants in *TXNIP*, previously described as causing a mitochondriopathy with hyperlactatemia, can also cause adult-onset cardiomyopathy, skeletal muscle weakness and hypertriglyceridemia that ranges from mild to severe depending on diet.

In our cohort, TXNIP deficiency most often resulted from different biallelic nonsense or missense variants in *TXNIP.* Nonsense variants led to complete absence of TXNIP protein in fibroblasts and decreased protein stability and proteasomal degradation were seen with the missense variant p.Asn203Tyr. Tissue-specific differences in protein stability and degradation may contribute to variability in organ involvement and clinical presentation among patients. We also identified a heterozygous 334 kb microdeletion within the TAR region in 1q21.1 [45,46], in combination with a nonsense variant in *TXNIP* in *trans* in two brothers. As the TAR syndrome is autosomal-recessively inherited, the brothers are not affected by TAR syndrome but TAR syndrome carriers. To our knowledge, no other disease than TAR syndrome has ever been reported with a TAR deletion before. Now, reporting that a TAR deletion on one allele unmasks a recessively acting *TXNIP* variant on the other allele, and compound inheritance causes autosomal-recessive *TXNIP*-associated disease, genetic testing for not only *RBM8A* but also *TXNIP* carrier status should be offered to partners of TAR deletion carriers. Of note: As the minimally 200 kb deleted region at 1q21.1 for TAR-syndrome is inherited from a clinically healthy parent in up to 75 % of individuals with TAR syndrome [45,46], and as individuals with TAR syndrome are not known to have metabolic disturbances, one can largely exclude a contribution of the TAR deletion itself to the observed phenotype in the two brothers carrying a TAR deletion as one pathogenic *TXNIP* variant.

We found increased glycogen accumulation, fatty acid synthesis markers and lipid accumulation in multiple patient specimens. Mechanistically, TXNIP is long since known to regulate the membrane availability of glucose transporters [9,10] and thus of cellular glucose influx. In mice, *Txnip* deficiency has been associated with increased cellular glucose influx leading to hypoglycemia [11] and increased glycogen storage in cardiac [26] as well as oxidative skeletal muscle [47]. In line with this, hypoglycemia in individuals #1, #4, #6, #7 and #8 as well as glycogen accumulation in patient's liver and muscle (individual #6) specimens can readily be linked to the mechanisms described in mice. Lactic acidosis in humans with biallelic *TXNIP* deficiency and in *Txnip*-KO mice might also be caused by increased glucose uptake, in combination with the mitochondrial dysfunction known in human [31] and murine [29,48] TXNIP deficiency. Elevated intracellular glucose levels drive glycolysis; in mitochondrial dysfunction, LDH activity is increased to regenerate NAD+, ultimately resulting in lactic acidosis. Besides glycolysis or glycogen synthesis, intracellular accumulated glucose intermediates can also be shuttled into DNL. Building on established knowledge about the role of TXNIP and its collaborative transcription factor partner ChREBP in murine hepatic DNL [[19], [20], [21], [22], [23], [24]], our work provides human data that suggest elevated hepatic DNL in TXNIP deficiency. This is supported by increased DNL-pathway gene expression in liver, consonant with hepatic lipid accumulation in a patient with biallelic variants in *TXNIP* who showed hepatomegaly and elevated transaminase-activity values (individual #6). Two additional patients with biallelic variants in *TXNIP* also presented with hepatomegaly; suggesting a shared mechanism. Pathological lipid accumulation was also observed on muscle biopsy of individual #6 and heart biopsy of individuals #1 and #2, confirming multi-organ involvement in *TXNIP*-associated lipid metabolism derangement. Increased DNL-pathway gene expression, increased triglyceride content and DNL-driven changes in the fatty acid saturation profile in muscle of individual #6 and in hearts of *Txnip*-KO mice further confirmed elevated DNL as the pathomechanism leading to pathological lipid deposition. These pathological lipid depositions were visible histopathologically in muscle of individual #6 and in hearts of individuals #1 and #2. These findings clinically may link to skeletal muscle weakness as observed in individuals #1, #3, #5, #6, #7, and #10, and cardiomyopathy as observed in individuals #1, #2 and #6. In sum, based on our results and in accordance with the literature [[19], [20], [21], [22], [23], [24]], it is tempting to speculate that increased DNL is part of the mechanism in TXNIP deficiency. As DNL in muscle is upregulated in obesity and known to cause insulin resistance, targeting elevated DNL is a promising therapeutic option in patients with biallelic variants in *TXNIP*. Although obtained in specimens from only three individuals with TXNIP deficiency, our findings align with metabolic observations in *Txnip*-KO mice sufficiently to permit us to propose increased DNL as causing organ lipid accumulation in TXNIP deficiency, potentially contributing to the clinical phenotype characterized by muscle weakness and cardiomyopathy. Variable expressivity of these features in our cohort remains to be further investigated; at the moment we observed an age-related association with cardiomyopathy in our small cohort.

Although TXNIP is known to control cardiac hypertrophy in mice in response to pressure overload and the TXNIP-interaction partner TXN is involved in pressure overload-induced cardiac hypertrophy [49], *TXNIP* variation has not previously been associated with human heart disease. In our study, we report adult-onset severe cardiomyopathy coupled with biallelic *TXNIP* variants in three of four adult individuals of whom two died from cardiomyopathy-associated complications. As the cardiac status of individual #4 was echocardiographically unremarkable at age 26, that expressivity of a cardiac phenotype in *TXNIP*-associated disease varies seems likely. Cardiac assessment in individuals with biallelic *TXNIP* variants appears urgent at latest in early adulthood; younger members of our study cohort have been offered such care but to date have declined. That adult individuals with TXNIP deficiency tend to show arterial hypertension (Table 1) suggests that early control of high blood pressure in TXNIP deficiency may be of special importance, even in children. As constitutive loss of TXNIP now has been associated with a potentially fatal cardiomyopathy, to include *TXNIP* variants in (virtual) panel analysis in genetic testing of patients with cardiomyopathy – especially those with concomitant lactic acidosis – should be considered.

Interestingly, individual #1 reported clinical improvement during dietary restriction of refined sugar intake, which he imposed on himself for several weeks every year. A mechanistic connection was suspected as a connection between diet and cardiac function had been shown in *Txnip*-KO mice, whose cardiac function was better preserved on a high-fat diet than is that of WT controls [30]. To determine whether increased lipogenesis in TXNIP deficiency is derived from dietary carbohydrates, and if this has any impact on the heart, we used a low-carbohydrate diet in *Txnip*-KO mice. Indeed, a low-carbohydrate diet could restore high muscle and heart lipogenesis rates in *Txnip*-KO mice to the lower rates in their WT littermates and reduce the TAG content in *Txnip*-KO mice hearts by roughly 20% while not changing TAG content in WT mice hearts. These findings indicate first that increased lipogenesis and TAG organ deposition in TXNIP deficiency is indeed at least partially derived from dietary carbohydrates, and second, that this pathomechanism is diet-responsive. The dietary association with reduced DNL transcription was observed in WT mice and preserved in TXNIP deficient mice. However, the reduction in heart TAG was seen only in TXNIP deficient mice. Therefore, we conclude that dietary interventions may open the possibility for further studies investigating a therapeutic diet option for individuals with biallelic *TXNIP* deficiency. Of additional importance was first, that low carbohydrate levels in the diet did not exacerbate hypoglycemia in mice (Figure 6K, as well as that diet-induced hypoglycemia did not occur in individual #1) and second, that the diet used was – compared to chow diet – hypercaloric to avoid any calorie restriction effects. However, low carbohydrate intake led to moderately higher plasma triglyceride levels in both *Txnip*-KO and WT mice. This effect thus also seems to be rather diet-dependent than TXNIP-dependent. Nonetheless, our findings suggest that in TXNIP deficiency, not only high fatty acid synthesis but also other mechanisms may contribute to increases in plasma TAG, such as impaired vascular catabolism of TAG-rich lipoproteins or the fact that consuming a low-carbohydrate diet implicates ingestion of larger amounts of lipids. Further, on a ketogenic diet – low carbohydrate intake with very high lipid intake, frequently used in patients with mitochondriopathies – individual #3 developed severe hypertriglyceridemia. To avoid high pancreatitis risks, dyslipidemia must be monitored strictly during dietary interventions in *TXNIP*-associated disease.

The association of TXNIP with hypertriglyceridemia in humans has long been debated [[50], [51], [52], [53], [54]]. Earlier studies concluded that single-allele variation in *TXNIP* does not cause an autosomal-dominant hypertriglyceridemia. Our work, however, demonstrates that an autosomal-recessive hypertriglyceridemia with variable expressivity and incomplete penetrance, most likely depending on additional environmental and dietary factors, results from biallelic variation in *TXNIP* and belongs to the broader phenotype of TXNIP deficiency. Therefore we conclude that *TXNIP* should be acknowledged as a candidate disease gene also for hypertriglyceridemia and needs to be tested in larger, independent cohorts. Variable expressivity and incomplete penetrance of hypertriglyceridemia were seen both in humans with biallelic TXNIP deficiency and in murine studies [25], which reported contradictory findings with respect to hypertriglyceridemia in *Txnip*-KO mice. In our study, *Txnip*-KO mice fed chow diet displayed fasting plasma triglyceride levels slightly higher than WT littermates. Possible reasons include variations in genetic background, diet composition, and experimental context.

Findings in TXNIP deficiency are of interest not only in respect of human genetic *TXNIP*-associated disease and of the clinical care for rare individuals with biallelic TXNIP deficiency, but also in their broader implications. This study addresses a disease state resulting from TXNIP absence. As TXNIP overabounds in common metabolic diseases such as diabetes mellitus and cardiovascular disease, our findings imply that consequences of abnormal TXNIP expression diverge depending on whether it is absent or elevated, and that TXNIP balance is needed for metabolic homeostasis. Further, as pharmacological inhibition of TXNIP protects against the development of diabetes in mice [16], TXNIP is an attractive therapeutic target. We suggest that potential short-term side effects of TXNIP-lowering pharmacological treatment may include hypoglycemia and lactic acidosis, as reflected in rapidly responding metabolic parameters. Hypertriglyceridemia might develop in the mid-term due to increased DNL. Cardiomyopathy and skeletal muscle weakness in human TXNIP deficiency on the other hand mechanistically are linked with glycogen and lipid accumulation and worsen over time. These accordingly might not be short- or mid-term side effects of a TXNIP-lowering drug therapy. Further investigation is needed in any event more completely to define the role of TXNIP in human metabolism and to understand how TXNIP dysregulation contributes to metabolic diseases, and how this knowledge can be leveraged to treat cardiometabolic disease.

Altogether, we describe TXNIP as a distinct modulator of human lipid and glucose metabolism in a tissue-specific manner, affecting the murine and human lipidome by regulating fatty acid synthesis. Biallelic variants in *TXNIP* are associated with a systemic disease affecting multiple organs and are linked to lactic acidosis, hypoglycemia, hypertriglyceridemia, adult-onset cardiomyopathy, and muscle weakness.

## 4 Methods

### Ethical statement

All analyses of patient information, plasma samples and muscle- and liver-biopsy specimens were carried out with written informed consent. Written informed consent for studies in children was obtained from their legally authorized representatives, with consent to publish identifiable information for patients and their parents. All studies were conducted in accordance with the Declaration of Helsinki and approved by the local medical ethics committees (Ethical Review Board of the Ärztekammer Hamburg: PV7038; Salzburg: Mitonet 415-E/1317/13–2020).

### 4.1 Animal model

All studies on animals were performed with permission of the Animal Welfare Offices of the University Medical Center Hamburg-Eppendorf and the Behörde für Gesundheit und Verbraucherschutz Hamburg. The *Txnip*-KO mouse line used was purchased from Jackson Laboratories. Mice were 8-36 weeks of age, and both males and females were studied. Mice were fed standard chow diet (16.2 MJ/kg, 67% kJ as carbohydrates) or Ssniff® E15660 (21.2 MJ/kg, 2% kJ as carbohydrates) and had *ad libitum* access to food and water under standard housing conditions. Dietary intervention with low carbohydrate-diet vs. chow diet was conducted for 15 days. Organ harvests were performed after a 4 h fasting period, and the mice were anesthetized with a lethal dose of ketamine and xylazine. Blood for glucose measurement was taken from the tail, while blood for ethylenediaminetetraacetic acid (EDTA) plasma measurements was taken by cardiac puncture. Collected organs were snap-frozen in liquid nitrogen pending analysis.

### 4.2 Plasma profiling

EDTA blood from humans and mice was centrifuged shortly after collection and plasma was frozen pending analysis. Blood plasma parameters were evaluated using the Cobas c 111 system (Roche).

### 4.3 Cell cultures

Fibroblast cultures were derived from patients or from age-matched adult and child controls. Cells were cultured at 37 °C in 5 % carbon dioxide in Dulbecco's Modified Eagle Medium with 4.5 g/L d-glucose, l-glutamine, and pyruvate (Gibco, Thermo Fisher Scientific). All media were supplemented with 10 % fetal calf serum (Sigma-Aldrich) and 1 % Anti-Anti (penicillin/streptomycin/amphotericin B; Gibco). To inhibit proteasomal activity, InSolution™ MG132 (Calbiochem, Cat.-No. 474791), 20 μM, was added to cell-culture supernatants for 6 h.

### 4.4 Gene expression

For RNA isolation, tissues were homogenized in TRIzol® (Invitrogen) using a TissueLyser (Qiagen). The homogenate was mixed with 200 μL of chloroform. The aqueous phase was mixed with 300 μL absolute ethanol and purified using a NucleoSpin RNAII Kit (Macherey-Nagel). RNA concentrations were measured using Thermo Scientific NanoDrop™ equipment. Quality was determined at 260/280 nm absorbance ratio. cDNA was synthesized using a High-Capacity cDNA Archive Kit (Applied Biosystems). Gene expression was measured using SYBR green (Applied Biosystems). The values were normalized to those for the indicated housekeeper.

### 4.5 Oral glucose tolerance testing

Mice fasted for 4 h were gavaged with glucose (2 g/kg body weight). Tail-vein blood was collected at indicated time points. Glucose levels were measured using Aviva AccuCheck glucose sticks (Roche). At 2 h, mice were anesthetized (xylazine/ketamine) and blood for plasma was collected by left ventricular puncture.

### 4.6 Histologic studies

For routine histologic work, harvested organs were fixed in a 4 % paraformaldehyde solution for at least 24 h. Samples were embedded in paraffin and sectioned. Sections stained with H&E and PAS techniques were examined by light microscopy. For ultrastructural study, TEM-fixed tissue was stained *en bloc* with osmium tetroxide (Science Services, Munich, Germany) and embedded in EPON-812 equivalent Agar 100 Resin (Agar Scientific). Semithin sections counterstained with azure 2/methylene blue were examined by light microscopy and ultrathin sections stained with uranyl acetate (Polyscience, Eppelheim, Germany) and lead citrate (Riedel-de Haën, Seelze, Germany) were examined using a Zeiss EM900 transmission electron microscope (Zeiss) and a 2k × 2k slow-scan CCD camera (Tröndle Restlichtverstärkersysteme).

For TEM of human muscle, samples were washed in 0.1 M cacodylate buffer (Sigma-Aldrich), incubated for 2 h in 1 % osmium tetroxide, dehydrated in an ascending series of ethanols, and embedded in Epon 812 (Serva). Ultrathin sections were counterstained with uranyl acetate and lead citrate, and examined using a LEO 912 AB OMEGA electron microscope (Leo Elektronenmikroskopie, Oberkochen, Germany).

### 4.7 Lipidome analyses

Lipidome analysis was performed using the Lipidyzer™ Platform from SCIEX. Plasma samples were spiked with Lipidyzer™ Internal Standards (SCIEX), following lipid extraction employing an adjusted 2-methoxy-2-methylpropane (MTBE)/methanol (MeOH) extraction protocol [18]. Lipids were concentrated and reconstituted in a 50 : 50 mixture of dichloromethane and MeOH containing 10 mM ammonium acetate. Differential mobility spectrometry and QTRAP® system processing (QTRAP® 5500; SCIEX) permitted separation and targeted profiling of lipid species. Lipids were quantified using Lipidyzer™ software (Lipidomics Workflow Manager; SCIEX) using specific multiple reaction monitoring transitions.

### 4.8 Mass spectrometry

For homogenization, tissue was transferred into a 2 mL sample tube. Lysis buffer (900 μL), HALT phosphatase inhibitor cocktail (Thermo Fisher), and a steel bead were added and the samples were homogenized in a bead mill (TissueLyser II, Qiagen, Hilden, Germany) for 2 min at 25 Hz. The samples were incubated for 5 min at 95 °C and 800 rpm, followed by ultrasonication with 5 pulses at 30 % power. Cold MTBE (750 μL) was added, and samples were shaken for 1 h at 4 °C. To induce phase separation, 188 μL of water with 0.1 % ammonium acetate was added. The extracts were then centrifuged at 10,000×*g* for 5 min, and the upper phase, containing lipids, was collected. To precipitate proteins, methanol (MeOH) was added to the remaining lower phase at a 4:1 v:v ratio MeOH:water). The mixture was incubated for 1 h at −20 °C and then centrifuged for 12 min (13,000×*g*) at 4 °C. The pellet was resuspended in lysis buffer. Bicinchoninic acid assays (Thermo Fisher) were performed for all samples following manufacturer's instructions. For proteomics samples, the single-pot solid-phase-enhanced sample-preparation (SP3) protocol was followed with 20 μg of starting material, as described above. For phosphoproteomics samples, 200 μg of starting material were used. After tryptic digestion, phosphopeptides were enriched using the High-Select Fe-NTA Magnetic Phosphopeptide Enrichment Kit (Thermo Fisher) according to manufacturer's instructions. Chromatographic separation of peptides was achieved using a two-buffer system (buffer A, 0.1 % formic acid [FA] in H_2_O; buffer B, 0.1 % FA in acetonitrile [ACN]) on a nano ultra-high-performance liquid chromatography system (UHPLC; Dionex Ultimate 3000 UHPLC system, Thermo Fisher Scientific). The system was equipped with a peptide trap (column) (100 μm × 20 mm, 100 Å pore size, 5 μm particle size, C18, Nano Viper, Thermo Fisher) for online desalting and purification, followed by an analytical C18 reversed-phase column (75 μm × 250 mm, 130 Å pore size, 1.7 μm particle size, peptide BEH C18, nanoEase, Waters). Peptides were separated using an 80 min method with a linear increase in ACN concentration from 2 % to 30 % over 60 min.

Tandem mass spectrometry (MS/MS) was performed on a quadrupole-ion-trap-orbitrap mass spectrometer (Orbitrap Fusion, Thermo Fisher Scientific). Eluting peptides were ionized using a nano-electrospray ionization source with a spray voltage of 1,800 V and analyzed in data-independent acquisition (DIA) mode. For each survey scan, ions were accumulated for a maximum of 240 ms or until an automatic gain control (AGC) target of 2 × 10^5^ ions. Fourier-transformation based mass analysis of the data from the orbitrap mass analyzer was performed covering a mass range of *m*/*z* 400–1,200 with a resolution of 120,000 at m/z = 200. Fragmentation in DIA mode with 12 *m*/*z* isolation windows with 1 *m*/*z* window overlaps was performed within a mass range of *m*/*z* 400–800. Fragmentation was carried out with a normalized collision energy of 30 % using higher energy collisional dissociation. An AGC target of 5 × 10^4^ ions and a maximum injection time of 54 ms was set. Orbitrap resolution was set to 30,000 with a scan range from *m*/*z* 350–2000.

Liquid chromatography-MS/MS data were analyzed using the Sequest algorithm integrated into Proteome Discoverer software (v3.1, Thermo Fisher Scientific) against a reviewed mouse database, obtained in November 2023 (17,289 entries), using Inferys 3.0 fragmentation as the prediction model. Carbamidomethylation was set as a fixed modification for cysteine residues. Oxidation of methionine was included as a variable modification. For phosphoproteomics samples, phosphorylation of serine, threonine and tyrosine was set as a variable modification. A maximum number of one missing tryptic cleavage was set. Peptides between 7 and 30 amino acids were considered. A strict cut-off (false discovery rate (FDR) < 0.01) was set for peptide identification. Quantification was performed using the CHIMERYS algorithm based on fragment ions. Protein abundances were log_2_-transformed and normalized to the column median before statistical analysis.

### 4.9 Western blot

Cultured cells were washed twice with cold phosphate buffered saline (PBS) and scraped off in 1 mL of PBS buffer. The mixture was centrifuged for 5 min at 650×*g* and 4 °C. The supernatant was removed and resuspended in 50 μL cell lysis buffer (50 mM Tris-HCl, pH 8.0; 150 mM NaCl; 1 % Nonidet P-40; 0.5 % Na-deoxycholate; 5 mM EDTA, 0.1% sodium dodecyl sulfate [SDS]) supplemented with complete Mini Protease Inhibitors and PhosStop (Roche). Organs were harvested and homogenized directly in supplemented lysis buffer. Clarification of cell lysates and organ lysates was performed by centrifugation for 10 min at 14,000 rpm and 4 °C. Supernatants were supplemented with sample buffer and proteins were separated on SDS-polyacrylamide gels and transferred to polyvinylidene fluoride membranes using the Transblot Turbo Transfer System (Bio-Rad Laboratories). Following blocking (20 mM Tris-HCl, pH 7.4; 150 mM NaCl; 0.1 % Tween-20; 5 % non-fat dry milk) and washing (20 mM Tris-HCl, pH 7.4; 150 mM NaCl; 0.1 % Tween-20), membranes were incubated in primary antibody solution (20 mM Tris-HCl, pH 7.4; 150 mM NaCl; 0.1 % Tween-20; 5 % BSA or 5 % non-fat dry milk) overnight containing the appropriate antibodies. Membranes were washed and incubated with donkey anti-rabbit IgG horseradish peroxidase secondary antibody (GE Healthcare; no. NA934V; 1:7,500 dilution) or with sheep anti-mouse IgG horseradish peroxidase secondary antibody (GE Healthcare; no. NA931V; 1:7,500 dilution) for 1 h. After final washing, protein signals were visualized using the ChemiDoc MP Imaging System (Bio-Rad Laboratories).

### 4.10 Exome/whole genome sequencing and variant validation

Genomic DNA extracted from blood samples was subjected to trio exome or whole genome sequencing. For exome sequencing, coding DNA fragments were enriched with a SureSelect Human All Exon 50 Mb V5 Kit (Agilent) and libraries were sequenced on a HiSeq2500 platform (Illumina). Reads were aligned to the human reference genome (UCSC GRCh38/hg38) using the Burrows-Wheeler aligner (v.0.5.87.5). Genetic variation was detected using SAMtools (v.0.1.18), PINDEL (v. 0.2.4t), and ExomeDepth (v.1.0.0). Impact of predicted amino acid substitutions on protein function was assessed by the pathogenicity prediction tools CADD, M-CAP, and ClinPred. For whole genome sequencing, simultaneous sequencing and primary data processing of libraries generated using the Illumina DNA PCR-free library preparation protocol were performed on a NovaSeq X (Illumina) platform, producing 2 × 150 bp paired-end reads. Quality control and preprocessing of the genomic data were performed on an Illumina DRAGEN system using DRAGEN software version 4.4 (Illumina). The raw sequencing data were aligned to the human reference genome GRCh38 (hg38). Detection of single-nucleotide variants (SNVs), copy number variants (CNVs), repeat-length variants, and structural variants was carried out using the manufacturer-recommended analysis tools implemented in the DRAGEN software. Interpretation of the identified sequence variants was performed using Emedgene software version 100.39 (Illumina). All reported variants were visually validated using the Integrated Genome Viewer (IGV). Impact of predicted amino acid substitutions on protein function was assessed by the pathogenicity prediction tools CADD, REVEL, AlphaMissense, GeneSplicer, MaxEntScan, NNSplice, SpliceAI, and SSF-like were used additionally. After both exome and whole genome sequencing, variants were validated by Sanger sequencing. Primer pairs were designed to amplify selected coding exons of the candidate gene. Amplicons were directly sequenced using an ABI BigDye Terminator Sequencing kit (Applied Biosystems) and a capillary sequencer (ABI 3500, Applied Biosystems). Sequence electropherograms were analyzed using Sequence Pilot software (JSI Medical Systems).

### 4.11 Statistical methods

Data are displayed as mean ± standard error of mean. Comparisons of two groups were examined using Student's *t*-test or the Mann-Whitney *U* test. Comparisons of three or more groups were analyzed using ANOVA. Perseus (Version 2.0.11.0), GraphPad Prism and Microsoft Excel were used for all statistical analyses.

## CRediT authorship contribution statement

**Julia-Josefine Scholz:** Conceptualization, Data curation, Formal analysis, Investigation, Methodology, Visualization, Writing – original draft, Writing – review & editing. **Sharlaine Y.L. Piel:** Conceptualization, Data curation, Formal analysis, Investigation, Methodology, Visualization, Writing – original draft, Writing – review & editing. **Ioannis Evangelakos:** Data curation, Formal analysis, Investigation, Methodology. **Christian Müller:** Formal analysis, Resources, Visualization. **Daniela Karall:** Investigation, Resources, Writing – review & editing. **Birgit Reinhart-Steininger:** Investigation, Resources, Writing – review & editing. **Katja Steinbrücker:** Investigation, Resources, Writing – review & editing. **Thomas Stulnig:** Investigation, Resources, Writing – review & editing. **Julia Rohde:** Investigation, Writing – review & editing. **Johannes Mayr:** Investigation, Writing – review & editing. **Matias Wagner:** Formal analysis. **Ali Biabani:** Data curation, Formal analysis, Investigation, Methodology, Writing – review & editing. **Thomas Mair:** Data curation, Formal analysis, Investigation, Methodology, Writing – review & editing. **Kilian Müller:** Formal analysis, Investigation, Methodology, Writing – review & editing. **Hartmut Schlüter:** Methodology, Resources, Writing – review & editing. **Sören Weidemann:** Formal analysis, Investigation, Methodology. **Viktoria Chirico:** Formal analysis, Investigation, Writing – review & editing. **Christina Weiler-Normann:** Investigation, Resources. **Christian Hagel:** Formal analysis, Investigation, Methodology, Writing – review & editing. **A.S. Knisely:** Formal analysis, Investigation, Methodology, Writing – original draft, Writing – review & editing. **Joerg Heeren:** Investigation, Resources, Writing – review & editing. **Benedikt Schoser:** Investigation, Resources, Writing – review & editing. **Christian Kubisch:** Investigation, Resources, Writing – review & editing. **Maja Hempel:** Investigation, Resources, Writing – review & editing. **Holger Prokisch:** Investigation, Resources, Writing – review & editing. **Saskia B. Wortmann:** Formal analysis, Investigation, Resources, Writing – review & editing. **Anna Worthmann:** Data curation, Formal analysis, Investigation, Methodology, Resources, Validation, Visualization, Writing – review & editing. **Christian Schlein:** Conceptualization, Data curation, Formal analysis, Funding acquisition, Investigation, Methodology, Project administration, Resources, Supervision, Validation, Visualization, Writing – original draft, Writing – review & editing.

## Declaration of competing interest

The authors declare that they have no known competing financial interests or personal relationships that could have appeared to influence the work reported in this paper.

## Data Availability

Data will be made available on request.
