# Supplementary material for: Biallelic TXNIP deficiency is associated with a multisystemic metabolic disease
Source: Mol Metab. 2026 Jul 14;111:102414. doi: 10.1016/j.molmet.2026.102414 (PMC13450574; doi:10.1016/j.molmet.2026.102414)
Supplement: Supplementary file 1 [file mmc2.pdf]

Supplementary Figure 1

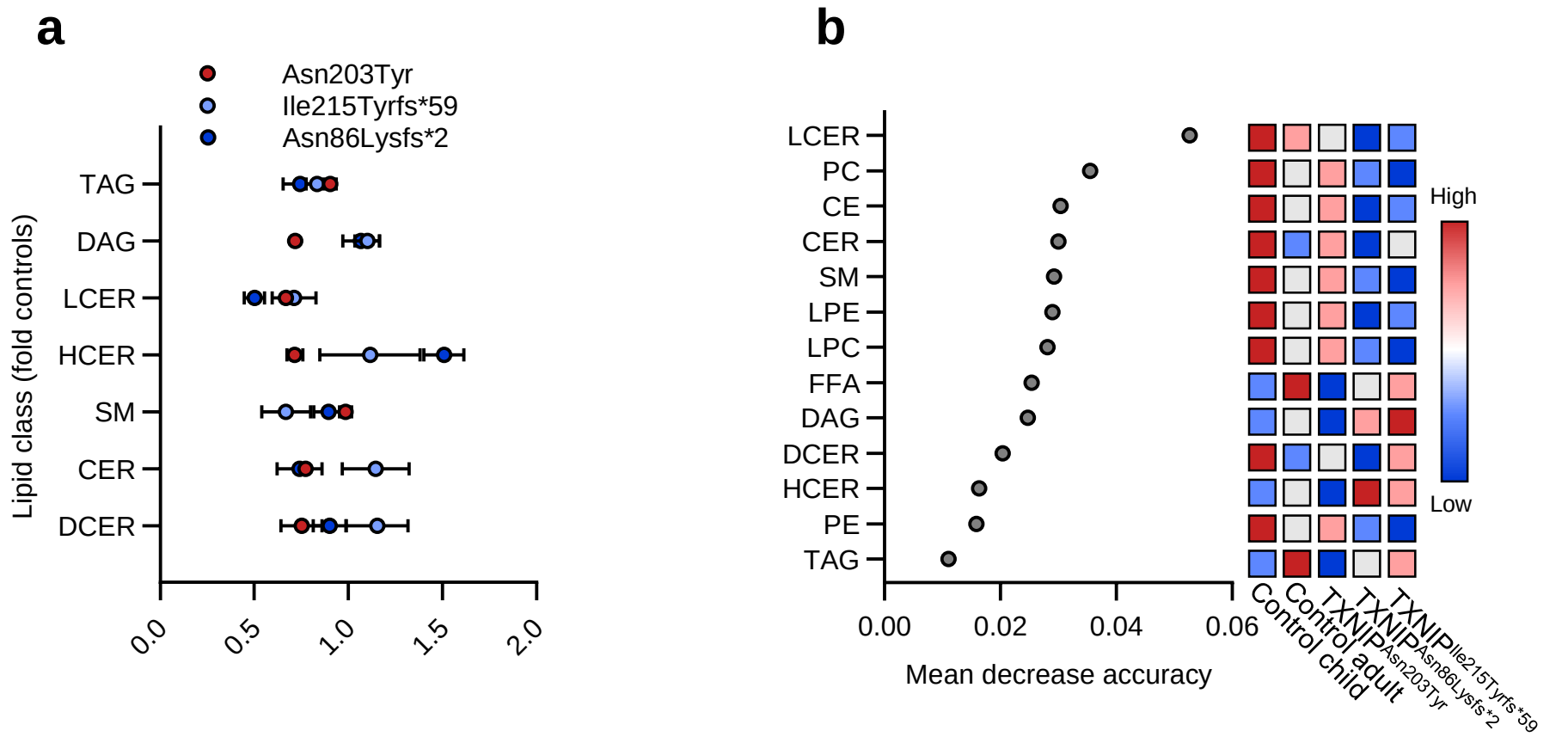

Supplementary Fig.1: (a) Relative distribution of lipid classes in TXNIP Asn203Tyr, Asn86Lysfs*2 and Ile215Tyrfs*59 fibroblasts. (b) Random forest plot of lipidome, TXNIP Asn203Tyr, Asn86Lysfs*2 and Ile215Tyrfs*59 fibroblasts, compared to healthy controls (n=5). TAG: triglycerides, DAG: diglycerides, LCER: lactosylceramides, HCER: hexosylceramides, SM: sphingomyelins, CER: ceramides, DCER: dihydroceramides.

Supplementary Figure 2

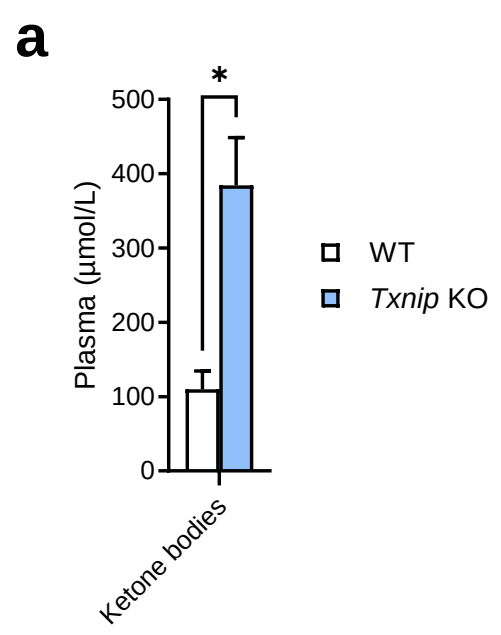

Supplementary Fig.2: (a) Plasma ketone bodies in *Txnip*-KO mice and WT littermates. WT mice n=11, *Txnip*-KO mice n=10. (g) WT mice n=5, *Txnip*-KO mice n=7.
