# Supplementary material for: Biallelic TXNIP deficiency is associated with a multisystemic metabolic disease
Source: Mol Metab. 2026 Jul 14;111:102414. doi: 10.1016/j.molmet.2026.102414 (PMC13450574; doi:10.1016/j.molmet.2026.102414)
Supplement: Supplementary file 2 [file mmc1.docx]

**Supplementary information**

| **Content** | **Page** |
| --- | --- |
| Medical history of individuals #1-7 | 1 |
| Supplementary references | 7 |

***Individual #1***

A 47-year-old man presented with cardiomyopathy of unknown origin, exercise intolerance, and muscle weakness. Recurrent hyperlactatemia and elevated serum creatine kinase activity levels (CK) were documented. Exercise intolerance had been present since youth. Cardiomyopathy was discovered at age 21 on routine check-up. Upper-extremity muscle weakness began at age 44. Lactate values were 14.2 mmol/L at maximum and 5.6 mmol/L at minimum. He reported a single hypoglycemic event (36 mg/dL) that required medical attention.

He was the second of three children born to healthy, non-consanguineous parents. The father had coronary heart disease, arterial hypertension, rheumatism, and leukemia. The medical history of the mother was unremarkable. A younger sister was healthy. An older brother (detailed medical history, see individual #2) died from acute decompensation of cardiac insufficiency aged 33 only days after diagnosis of a previously unrecognized cardiomyopathy.

The individual's childhood development was unremarkable. Exercise intolerance became evident during physical education in school. Despite regular exercise, the individual had difficulties building endurance and muscle strength. He reported that earlier testing had shown slightly elevated serum CK-MB values. These were not investigated. His cardiomyopathy was of mixed hypertrophic and dilated form. During follow-up, ejection fraction (EF) aged 30 was 31% at minimum, leading to initiation of treatment for cardiac insufficiency. Aged 44, permanent chest pain developed, accompanied by muscle pain and weakness of upper-extremity muscles. At presentation, echocardiography showed improved EF to 41% upon heart failure therapy as well as moderate left ventricular hypertrophy. He also had left bundle branch block and arterial hypertension. On tertiary-care referral, CK-MB (404 U/L) and lactate (10.2 mmol/L) values were elevated. During cardiac stress test, lactate values rose to 14.2 mmol/L. Cardiac stress magnetic resonance imaging found no signs of myocardial stress-induced ischemia. Histologic examination of endomyocardial-biopsy material found hypertrophied myocytes, without disarray, and mild interstitial fibrosis, changes interpreted as non-specific. Hyperlactatemia prompted ultrastructural study for evidence of mitochondriopathy. Mitochondria were abnormal in configuration and size, with slightly irregular cristae.

Exome sequencing revealed a 334 kb TAR (thrombocytopenia – absent-radius syndrome) deletion in 1q21.1, spanning *TXNIP*, on one allele and a *TXNIP* c.253G>T, p.Glu85* nonsense variant on the other allele, indicating biallelic TXNIP deficiency.

Besides cardiac and muscular issues, the individual suffered from memory difficulties. Since age 45, he has sometimes forgotten appointments and the names of long-known colleagues and friends. He reports gender dysphoria and has been diagnosed as infertile owing to abnormal spermatogenesis.

The individual self-initiated a sugar-free diet for several weeks per year with self-reported increase in well-being, muscular strength and mobility. The hypoglycemic event (*v.s*.) did not occur while on a sugar-free diet.

***Individual #2***

The older brother of individual #1, individual #2, died aged 33 in multi-organ failure following acute heart failure caused by previously undetected cardiomyopathy. Genetic testing revealed the variants found in individual #1: A 334 kb TAR deletion in 1q21.1, spanning *TXNIP*, on one allele and a *TXNIP* c.253G>T, p.Glu85* nonsense variant on the other allele.

He described himself as healthy before the onset of acute illness, with self-referral to an emergency room for chest pain. Echocardiography revealed a substantially reduced biventricular EF (10%) and a thrombus in the dilated left ventricle. Initial blood-gas analysis found a lactate level of 15 mmol/L. On the same day he was admitted to a cardiac-surgery intensive care unit (ICU) where a left ventricular assist device was placed and veno-arterial extracorporeal membrane oxygenation (ECMO) for right ventricular assistance was begun. Acute renal failure was treated with veno-venous hemodialysis. Lactate values rose to > 31 mmol/L, leading to severe metabolic acidosis that could be compensated by tris-buffer administration only in part. In the course of ICU treatment, computerized tomography revealed cerebrovascular hemorrhage, with signs of elevated intracranial pressure. This was treated by hemicraniectomy. Multi-organ failure ensued, with persistent lactatemia and global hypoperfusion of the brain. Death supervened seven days after initial presentation, when supportive care was withdrawn. Histologic examination of cardiac tissue biopsied during support-device placement surgery found hypertrophied myocytes, endocardial fibrosis, diffuse interstitial fibrosis and inflammatory infiltrates. Further, parvovirus B19 DNA was detected, indicating that concurrent active myocarditis might have aggravated his underlying severe cardiomyopathy.

***Individual #3***

This boy was the fourth child of consanguineous parents. His three siblings and his parents were well. During pregnancy, corpus callosum agenesis and signs suggesting hydrops fetalis were noted. He was born at 38+2 weeks by cesarean section due to breech position. Generalized hypotonia, hepatomegaly, and facial dysmorphism were noted at birth, as were a cataract of the left eye and a patent *foramen ovale* without hemodynamic consequences. Lactic acidosis noted at birth (max 9 mmol/L, ref < 2 mmol/L) decreased after the neonatal period to 3-4 mmol/L. Two attempts at 3 : 1 ketogenic dietary therapy at ages four months and 13 months were stopped due to hyperlipidemia. At last follow-up aged six, he exhibited generalized hypotonia and severe delay in all areas of development. He could walk ~ 300 meters with assistance and communicated orally with sounds and a handful of meaningful words. He was a friendly and interactive child with no behavioral issues.

***Individual #4***

This woman, born in 1994, first presented at age 26 with an episode of abdominal pain and emesis after a fasting period. Ketotic and lactic acidosis accompanied hypoglycemia. Fasting serum triglyceride values were elevated (325 mg/dL, ref. <150 mg/dL), as were serum levels of creatinine (1.87 mg/dL) and uric acid (13.4 mg/dL). CK values were not elevated. Echocardiography showed no signs of hypertrophic cardiomyopathy or impaired ventricular function. Symptoms as well as lactic acidosis and ketosis resolved with parenteral glucose infusion. Serum triglycerides remained slightly elevated (189 mg/dL, ref. <150 mg/dL), but kidney function normalized. Several months earlier, she had been admitted to hospital elsewhere with similar symptoms and was similarly treated. She had been diagnosed with essential arterial hypertension aged 16.

She suffered from multiple calcium oxalate kidney stones. Previous investigation had identified chronically elevated CK values (400-500 IU/L) and asymptomatic low glucose levels (~ 45 mg/dL). Serum transaminase activities were within normal ranges and serum lipid values fluctuated between normal and moderately elevated levels. The individual’s two siblings and their mother were healthy. No information was available on her father.

Childhood development was unremarkable. She repeatedly complained of muscle weakness, but neurologic examination identified no abnormality. An electromyographic study was not performed. Self-reported hearing impairment was not confirmed by an otolaryngologist. Exome sequencing revealed a biallelic frameshift variant (c.[257dupA]; p.Asn86Lysfs*2) in *TXNIP*.

Use of a subcutaneous glucose sensor revealed prolonged low glucose levels, especially during night-time. The individual was advised to take 40 g of starch before going to bed as well as to avoid longer fasting periods. Two additional episodes of ketotic and lactic acidosis occurred after fasting or during illness. Annual monitoring found persistent slight elevations in levels of lactate, serum lipids (mostly LDL cholesterol), and CK.

***Individual #5***

Individual #5, a boy, is the first child of healthy consanguineous parents. Abnormal cardiotocography prompted emergency cesarean section at 37+3 weeks of gestation. Birth weight, length and head circumference were within normal ranges. Postnatal adaptation and the first four weeks of life were unremarkable. The parents noticed feeding difficulties, increased crying and a decline in general well-being at age five weeks.

Persistent lactic acidosis was observed during a hospital stay and during outpatient care thereafter, without hypoglycemia or dyslipidemia. Suspected mitochondriopathy was treated with mitochondria-supportive medication (“mito-cocktail”) including coenzyme q10, riboflavin, thiamine and carnitine, discontinued by the parents subsequently as too difficult to administer.

Echocardiography showed a persistent foramen ovale with otherwise unremarkable cardiac structures and function. Brain sonograms were unremarkable. Whole-genome sequencing identified a homozygous frameshift variant c.257dupA, p.Asn86Lysfs*2 in *TXNIP*. At age four months the boy was thriving but showed mild developmental delay and muscular hypotonia.

***Individual #6***

A 25-year-old man presented with progressive exercise-induced muscle weakness, arterial hypertension and hypertrophic cardiomyopathy. Muscular symptoms began in early adulthood, while cardiovascular issues had been present since late childhood. He was the second of four children born to healthy consanguineous parents. His father died aged 60 of cardiomyopathy of undetermined etiology. His mother had lost one pregnancy to spontaneous miscarriage (month three) and another to stillbirth (month eight) but was otherwise in good health. His three sisters were healthy. He had five healthy children.

His childhood development was unremarkable. The first symptom reported was tachycardia at age 13; elevated blood pressure was identified, with echocardiography revealing a bicuspid aortic valve and mild aortic-valve insufficiency. At age 18, hypertrophic cardiomyopathy and arterial hypertension were diagnosed. Antihypertensive medication was administered. A few years later, he noted a gradual decline in muscle strength, with progressively increasing weakness in hands and legs for approximately one year before initial presentation aged 25. He did not then report cramps or impairment of sensation; cramps in his hands and legs developed later, with tingling paresthesia. He described a sensation of dizziness and fainting on several occasions. Blood glucose values were repeatedly within the low-normal or hypoglycemic range. A clinical fasting test showed asymptomatic hypoglycemia (glucose 52 mg/dL). Two seizures occurred, both in the context of hunger or stress. CK and lactate values were chronically high (respectively 500-1200 IU/L and 3.5-5 mmol/L), with lactic acidosis. Transaminase activities and plasma lipid values fluctuated between normal and moderately elevated levels.

A muscle biopsy revealed excessive accumulation of both glycogen and abnormal lipid deposits. A liver biopsy showed moderate accumulation of both lipids and glycogen, as well as irregular mitochondrial structure. Exome sequencing revealed the biallelic variant c.607A>T, p.Asn203Tyr, in *TXNIP*.

At age 25, a knee cyst was surgically removed. Episodes of gout beginning at age 25 were treated with allopurinol.

At age 32, occlusion of the popliteal arteries by thrombus embolized from within the left ventricle presented as critical ischemia of both legs. He underwent intraluminal lysis and embolectomy. Impaired wound healing was successfully treated with vacuum-assisted closure. Warfarin was begun to provide anticoagulation. During this hospitalization, lactate and CK levels were high (respectively 3.6-10.5 mmol/L and 352-651 IU/L).

Cardiac function remained stable for years, with slight to moderate impairment observed. At age 35, he presented with palpitations, chest pain, and dyspnea. An absolute tachyarrhythmia (TAA) accompanied markedly reduced cardiac function, characterized by an EF of 10–15%, with acute decompensation manifest as low-output failure. Administration of the positive inotropic agent levosimendan permitted cardiac recompensation and stabilized cardiac function. The TAA was treated by electrocardioversion, followed by intensification of his pharmacologic heart-insufficiency regimen. During this hospitalization, serum lactate levels ranged between 3.4 and 7.6 mmol/L. One month later, TAA recurred. It was again treated by cardioversion. Amiodarone was begun, but was withdrawn due to QTc-time prolongation. Pulmonary-vein isolation was performed to prevent further TAA. As cardiac function continued to deteriorate (EF 10%) and lactate levels ranged between 6.0 and 11.7 mmol/L, recurrence of low-output heart failure was suspected. Levosimendan administration did not lower lactate levels. He complained of nausea and discomfort; elevated transaminase activities and spontaneous dyscoagulation prompted suspicion of liver-function impairment. Liver sonography revealed no parenchymal damage. A slight increase in kidney retention parameters indicated impaired renal function. Metabolic decompensation was hypothesized. Stabilization was observed upon administration of fluids, parenteral alimentation and coenzyme Q10, with improvements in organ function parameters as well as in coagulation. Concentrations of lactate also fell (4.1 mmol/L). During this hospitalization, CK levels were elevated (571 IU/L). Cardiac function recompensated but did not improve with medical treatment, so implantation of a cardiac resynchronization therapy (CRT) defibrillator was scheduled to prevent sudden cardiac death and to improve cardiac function. Evaluation for heart transplantation was offered but was declined on religious grounds. During CRT-device implantation, he once again developed arrhythmia, with a cardiac arrest shortly after the intravenous induction of anesthesia (fentanyl / propofol). Resuscitative measures included implantation of a mechanical cardiac support pump and initiation of ECMO. Progressive cardiogenic shock nonetheless developed, with death from multi-organ failure several days later.

***Individual #7***

This boy, born in 2014, is the first child of healthy consanguineous parents. Parts of his medical history have been described recently.^1^ Pregnancy was complicated by polyhydramnios. Due to breech position, cesarean section was performed at 38+6 weeks gestational age. Aged seven days, he was transferred from the birth hospital to a tertiary-care hospital because of floppiness, recurrent hypoglycemia and lactic acidosis. In his first three postnatal days, the lowest measured concentration of glucose was 30 mg/dL, with lactate values around 6.5 mmol/L and pH 7.11, thus a mitochondrial disorder was suspected. Also, hypertriglyceridemia (≤ 364 mg/dL) was noted. He was stable in the first three days after admission in transfer. At age ten days, pCO2 levels rose as high as 70 mmHg (due to bradypnea) and metabolic acidosis developed. Glucose was given by vein (10 mg/kg/min) to stabilize blood glucose concentrations. Lactate concentrations also stabilized but remained elevated at ~ 3-4 mmol/L. Muscular hypotonia did not resolve. On postnatal day twelve, liver and muscle biopsies were conducted, as was a skin punch biopsy for cultivation of fibroblasts. On postnatal day 20, glucose and lactate concentrations were stable on regular feeds. He was transferred to a peripheral hospital.

Exome sequencing of DNA from peripheral blood leukocytes revealed homozygosity for a nucleotide insertion in *TXNIP* (c.642_642insT, p.I215Yfs*59) causing a frameshift with a premature stop codon after 59 amino acids and yielding a truncated TXNIP variant. Each parent was heterozygous for the same mutation.

Psychomotor development was delayed, with increasingly frequent epileptic seizures beginning at age 18 months (up to 3-4 per month, controlled with levetiracetam, topiramate and lamotrigine). In response to stress, such as infections, the individual suffered from recurrent episodes of hypoglycemia. He was metabolically stable when this report was compiled, with marked developmental delay that has autistic features.

**Individuals #8, #9, #10**: Adapted from Katsu-Jimenez et al.^2^

**Supplementary References**

1.	Kahlhofer J, Marchet N, Zubak K, Seifert B, Hotze M, Egger-Hörschinger AS, Kucej L, Manzl C, Weyer Y, Weys S, Offterdinger M, Herzog S, Reiterer V, Volani C, Kwiatkowski M, Wortmann SB, Nemati S, Mayr JA, Zschocke J, Radlinger B, Thedieck K, Kremser L, Sarg B, Huber LA, Farhan H, de Araujo MEG, Kaser S, Scholl-Bürgi S, Karall D, Teis D. TXNIP mediates LAT1/SLC7A5 endocytosis to limit amino acid uptake in cells entering quiescence. EMBO J. 2025 Dec;44(23):7119-7153. doi: 10.1038/s44318-025-00608-9. Epub 2025 Oct 20. PMID: 41116060; PMCID: PMC12669767.

2.	Katsu-Jimenez, Y., Vazquez-Calvo, C., Maffezzini, C., Halldin, M., Peng, X., Freyer, C., Wredenberg, A., Gimenez-Cassina, A., Wedell, A., and Arner, E.S.J. (2019). Absence of TXNIP in humans leads to lactic acidosis and low serum methionine linked to deficient respiration on pyruvate. Diabetes *68*, 709-723. 10.2337/db18-0557.
